# The influence of hepatic arterial blood flow rate on holmium microsphere distribution: an MRI study in perfused porcine livers

**DOI:** 10.1186/s41747-025-00609-7

**Published:** 2025-08-06

**Authors:** Tess J. Snoeijink, Anne van den Brekel, Jan L. van der Hoek, Jaap G. M. Greve, H. Remco Liefers, Milou Boswinkel, Simon J. S. Ruiter, Joey Roosen, Erik Groot Jebbink, J. Frank W. Nijsen

**Affiliations:** 1 Radboud University Medical Centre, Department of Medical Imaging, Nijmegen, The Netherlands; 2 University of Twente, TechMed Centre, Multi-Modality Medical Imaging Group, Enschede, The Netherlands; 3 University of Twente, TechMed Simulation Centre, Enschede, The Netherlands; 4 University of Groningen, University Medical Centre Groningen, Department of Hepato-Pancreato-Biliary Surgery and Liver Transplantation, Groningen, The Netherlands

**Keywords:** Hepatic artery, Holmium, Microspheres, Perfusion, Swine

## Abstract

**Background:**

Transarterial radioembolisation (TARE) is a treatment for liver malignancies, involving the injection of radioactive microspheres in the hepatic artery (HA). Tumour-to-nontumour uptake varies among patients, possibly influenced by patient-specific blood flow profiles. To examine the impact of HA blood flow rate and high microsphere dosages on microsphere distribution in normal liver parenchyma, *ex vivo* magnetic resonance imaging (MRI)-guided machine perfusion experiments were conducted in porcine livers.

**Materials and methods:**

Porcine livers were subjected to oxygenated normothermic machine perfusion at three HA flow rates (0.02, 0.15, and 0.22 mL/min/g liver tissue; *n* = 3 per condition). Five fractions of 250 mg nonradioactive ^165^Ho-loaded microspheres were administered to *n* = 9 livers, and four additional fractions of 1,000 mg to *n* = 6 livers. Dynamic contrast-enhanced and Ho-sensitive T2*-weighed MR scans were acquired to extract perfusion rates, fictive dose maps, and homogeneity indices (HI).

**Results:**

Microsphere distribution correlated moderately with perfusion rate at low HA flow rate (*r* = 0.611), and very strongly at higher HA flow rates (*r* = 0.977 and 0.951 for 0.15 and 0.22 mL/min/g, respectively). Homogeneity increased with increasing flow rates, with HIs ranging from 3.68–4.72 at low, to 2.01–2.66 at medium, and 1.60–2.36 at high HA flow rate. HI decreased with higher microsphere concentrations, though distribution patterns remained unchanged.

**Conclusion:**

In our *ex vivo* model, higher HA flow rates resulted in more homogeneous microsphere distributions. The impact on tumourous tissue needs further investigation to determine whether pre-TARE HA blood flow measurements could improve microsphere distribution predictions.

**Relevance statement:**

Mapping of the hepatic arterial blood flow rate before transarterial radioembolisation and adjusting the treatment accordingly may help to improve outcomes for patients with liver cancer.

**Key Points:**

- Parameters influencing microsphere distribution were studied in MRI-perfused healthy porcine livers.
- Higher hepatic arterial blood flow rates led to more homogeneous microsphere distributions.
- Administering large numbers of microspheres did not alter microsphere distribution patterns.
- Impact on tumour tissue should be further investigated.

**Graphical Abstract:**

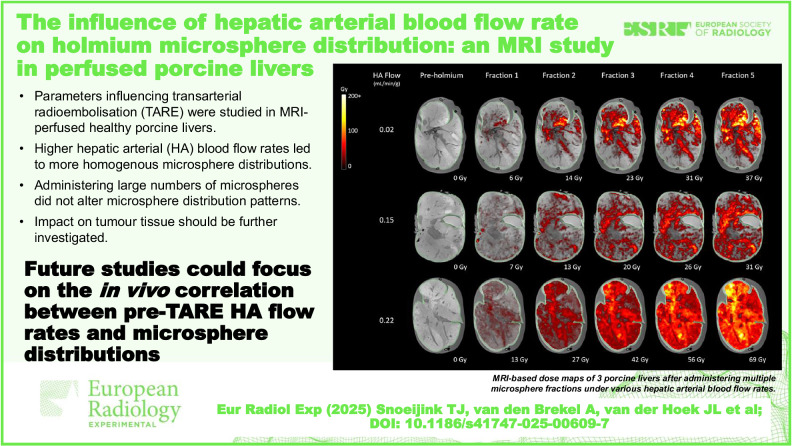

## Background

Transarterial radioembolisation (TARE) is a treatment method for both primary and secondary liver tumours [1, 2]. During TARE, radioactive microspheres are injected into the hepatic artery (HA) via a microcatheter. The HA blood flow will transport the microspheres predominantly towards the hepatic arterioles supplying the tumours. This technique relies on the liver’s unique dual blood supply, where tumour tissue is primarily supplied by the HA, while normal liver parenchyma receives most of its blood supply via the portal vein (PV). Three types of microspheres are commercially available, two contain the radionuclide ^90^Y, and a newer type contains the radionuclide ^166^Ho [3]. Although TARE is widely used, examining the parameters that influence microsphere distribution could aid in further optimising tumour-to-nontumour ratios, thereby improving survival and disease control [4, 5].

*In vitro* and *in silico* studies have shown that the degree of mixing between microspheres and blood plays an important role in microsphere distribution patterns [6, 7]. As stated by Aramburu et al [6], the higher the ratio between microsphere injection rate and blood flow rate, the more microspheres will cross blood flow streamlines. Alteration of the systemic flow rate, due to, for example, increased cancer burden, also leads to alterations in the ratio between microsphere injection rate and blood flow rate [6]. While *in vitro* and *in silico* studies have examined microsphere mixing dynamics, no studies have systematically investigated how flow rate variations influence microsphere distribution, even though it is well known that the HA flow rate is highly variable in diseased livers (220–813 mL/min [8, 9]). An additional uncertain parameter in TARE is whether high concentrations of microspheres can influence distribution patterns, potentially leading to HA stasis or backflow. In current clinical practice, this limitation may restrict the number of microspheres that can be safely administered [10].

To study the influence of HA flow rate and the influence of administering large numbers of microspheres on microsphere distribution, we propose *ex vivo* machine perfusion of healthy porcine livers. *Ex vivo* machine perfusion offers the capacity to maintain stable perfusion of *ex vivo* organs, allowing for controlled and systematic investigation of the behaviour of microspheres in the liver during radioembolisation. By performing the machine perfusion under magnetic resonance imaging (MRI), we can make use of the high-resolution MRI-based dosimetry properties of Ho microspheres.

This explorative study aims to investigate the impact of the HA blood flow rate on the distribution of microspheres within the porcine healthy liver parenchyma, using an MRI-compatible *ex vivo* machine perfusion model. A secondary aim is to investigate microsphere distribution patterns following the administration of large numbers of microspheres to a porcine liver.

## Materials and methods

### Liver procurement

Porcine livers were obtained from a local slaughterhouse. The procurement process did not interfere with the standard abattoir protocol and was in accordance with the Dutch Food Safety Authority guidelines. The complete organ package was requested, and the liver was isolated from the surrounding organs, for a detailed overview of the protocol see Supplemental #1.

The PV was cannulated with a 25 Fr cannula, and the inferior vena cava was incised for outflow. Through the PV cannula, 1 L cold 0.9% saline solution (Baxter International), containing 25,000 IU heparin (Heparin LEO 5,000 IU/mL, LEO Pharma) was administered, followed by another 1.5 L of cold saline solution. The interval between exsanguination and cold flush initiation was maintained under 30 min, as recommended in the literature [11]. Simultaneous to the liver harvest procedure, a 10 L canister was prepared with 30 mL 150,000 IE heparin, 60 mL 0.9% saline solution and 18 mL 50% glucose (B. Braun Medical) [12], which was then filled with non-autologous porcine blood.

In the laboratory, the HA was bluntly dissected up to the point of division into five branches supplying each liver lobe, with ligation of branches proximal to this level. The aorta was cannulated with a 25-Fr cannula; however, if the aorta was not intact, the HA was directly cannulated with a 12-Fr cannula. The superior vena cava was clamped. The liver was weighed before and after perfusion (Hendi weighing scale).

### Normothermic oxygenated machine perfusion

The Liver Assist device (XVIVO) with flow-driven software was used for perfusion through the PV using continuous flow. Perfusion through the HA side was performed using the Xenios NovaLung iLA ACTIVVE console (Medos Medizintechnik) with a DP3 pump head, generating pulsatility in the form of sine waves with an amplitude of 20% at 60 beats/min. The original Liver Assist disposable set with an oxygenator and Liver Assist heat exchanger was used. The oxygenator was supplied with compressed air (21% oxygen, 78% nitrogen) at a rate of 500 mL/min. Blood flow rates were measured on both the PV and HA sides using Sonotec clamp-on flow sensors (Sonoflow CO.55 V2.0/140), which were calibrated for use with blood. Perfusion was started at room temperature (21 °C) for 15 min with HA and PV flows at 50 and 150 mL/min, respectively. After that, the temperature was gradually increased by 1 °C per 2 min [13], towards normothermic porcine level, *i.e*., 38 °C [14]. Accordingly, the HA and PV flows were slowly increased to the flow rate of interest.

**Fig. 1:**
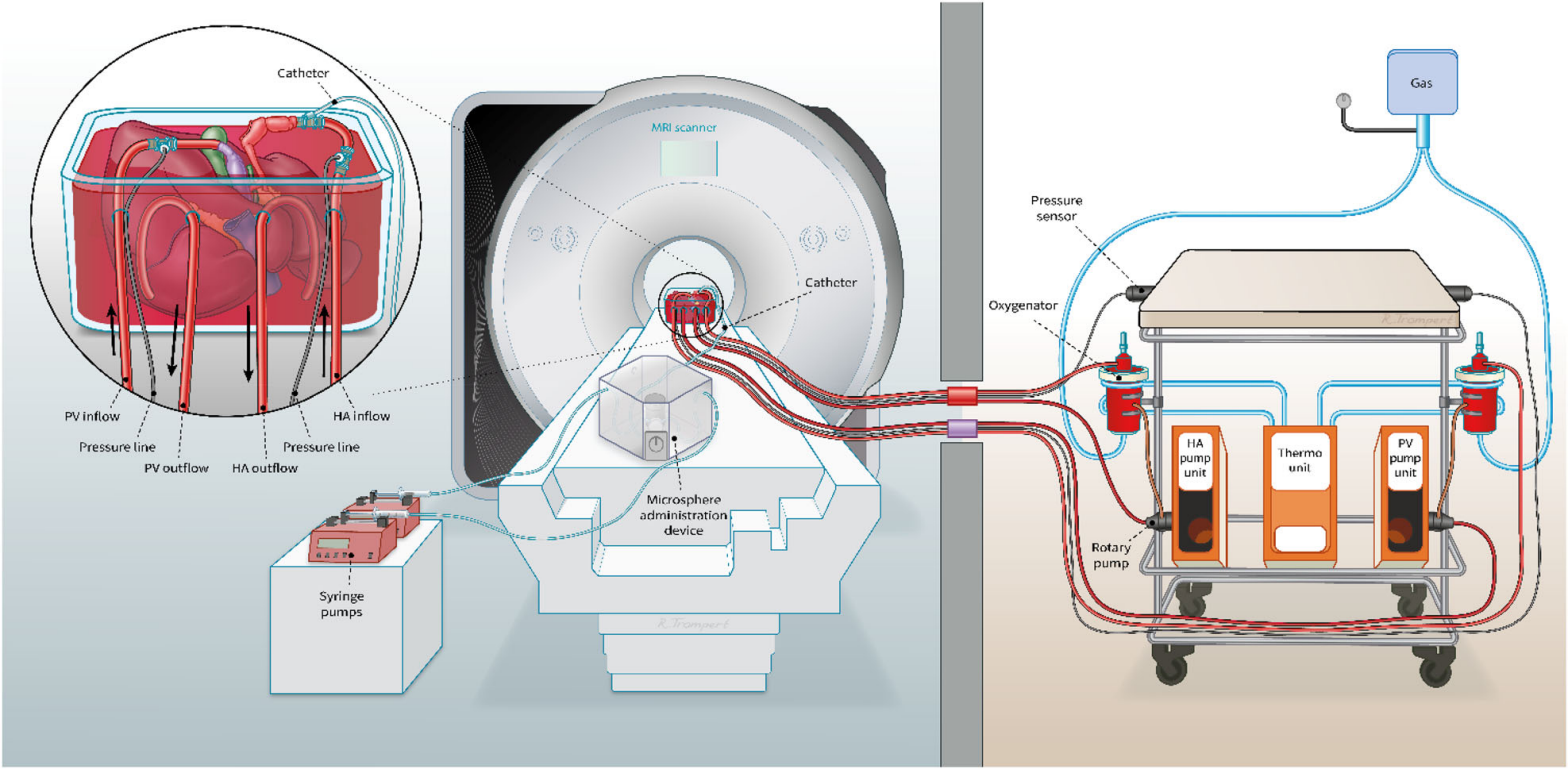
Schematic presentation of the MRI-compatible *ex vivo* machine perfusion setup. The liver is positioned inside the scanner (left) and is connected via eight metre polyvinylchloride tubing to the PV and HA pump units in the MRI control room (right). HA, Hepatic artery; PV, Portal vein; MRI, Magnetic resonance imaging

The liver was positioned inside a custom-modified MRI-compatible organ chamber filled with porcine blood. The organ chamber was connected to the pumping devices located in the MRI control room with eight metres of polyvinylchloride tubing (BRAND® special laboratory tubing, Sigma-Aldrich), with an inner diameter of ¼ inch (6.35 mm). Perfusion pressure was monitored at the height of the HA and PV cannula, through 4 × 210 cm of connected pressure lines (Edwards™ Lifesciences Corporation) using two sphygmomanometers (Durashock DS54, Welch Allyn). A schematic drawing of the setup is shown in Fig. 1.

### Investigated blood flow rates

Reported flow rates for human diseased livers (220–813 mL/min [8, 9]), when corrected for liver weight [15, 16], correspond to 0.15–0.57 mL/min/g of liver tissue. An extremely low HA flow rate condition (0.02 mL/min/g liver tissue) and two clinical representative HA flow rate conditions (0.15 and 0.22 mL/min/g liver tissue) were investigated, with corresponding PV flow consistently maintained at three times the HA flow rate [13]. The representative HA flow rates were based on reported flow rates from previous *ex vivo* porcine studies (Supplemental #2). Each condition was tested in *n* = 3 livers, resulting in a total of *n* = 9 livers included in this study.

### MRI

All imaging was performed using a 1.5-T MRI (MAGNETOM Aera, Siemens) with an 18-channel body array coil and a 32-channel spine coil. Details on the MRI sequences can be found in Table 1. After reaching the desired HA and PV flow rates, anatomical T1- and T2-weighted MRI scans were acquired. Then, perfusion quality was assessed by acquiring dynamic contrast-enhanced MRI scans. A gadolinium-based contrast agent (Dotarem® 0.5 mmol/mL) was administered (6 mL, 0.3 mL/s) while acquiring 200 volumetric series at an interval of 0.86 s. Subsequently, Ho-sensitive T2*-weighted scans were made before and after administering fractions of Ho microspheres.

**Table 1:** Details of the sequences used during *ex vivo* machine perfusion experiments under MRI in porcine livers. MRI Magnetic resonance imaging.

Name	Sequence type	Echo time (ms)	Repetition time (ms)	Flip angle (°)	Slice thickness (mm)
T1-weighted	Spin-echo	13	634	81	4
T2-weighted	Turbo spin-echo	96	5,800	150	4
T1 VIBE Dixon	Ultrafast spoiled gradient echo	2.39	6.75	10	1.1
Dynamic angiography	FLASH	0.86	2.2	25	8
T2*-weighted-Ho	Multiecho gradient echo (10 echoes)	1.7 (∆TE: 1.35)	1,040	90	4

*FLASH* Fast low angle shot, Δ*TE* Echo time increase, *VIBE* Volumetric interpolated breath-hold examination

### Microsphere administration

Five fractions of 250 mg nonradioactive ^165^Ho-loaded microspheres (30.0 µm, 1.4 g/mL [3]) were administered to each liver using the conventional administration device with a V-vial delivery system (Quirem Medical B.V.). To each vial, 2 mL 0.1% phosphate-buffered pluronic solution was added to facilitate microsphere agitation. Two syringe pumps (NE-4000 Multi-Phaser, New Era Pump Systems Inc.) were used for reproducible injections, with 3-metre polyurethane tubing (inner diameter of 0.4 mm) connecting the pumps outside the 5 gauss safety zone to the administration device. Injections were administered in pulses (0.1 mL per pulse) following the instructions for use for QuiremSpheres™ [17], at a rate of 32 mL/min with a 0.3-s pause between pulses to create sufficient microsphere agitation within the V-vial. This injection rate was selected while accounting for pressure loss caused by the extended tubing. Injections were performed via a 130-cm 2.7-Fr microcatheter (Progreat, Terumo Europe), with the catheter placed at least two centimetres before the bifurcation into the left and right HA.

### Image analysis

#### Perfusion rates

The dynamic contrast-enhanced MRI scans were used to determine the perfusion rate. The T1 VIBE DIXON anatomical scan was resampled to match the dimensions of the dynamic scan, and regions of interest were delineated for each liver lobe using a custom Matlab acquisition code (Matlab R2022a, MathWorks). Time-intensity curves were calculated using the percent enhancement formula

$${{\rm{P}}}{{\rm{e}}}{{\rm{r}}}{{\rm{c}}}{{\rm{e}}}{{\rm{n}}}{{\rm{t}}}\,{{\rm{e}}}{{\rm{n}}}{{\rm{h}}}{{\rm{a}}}{{\rm{n}}}{{\rm{c}}}{{\rm{e}}}{{\rm{m}}}{{\rm{e}}}{{\rm{n}}}{{\rm{t}}}=100\ast ({S}_{1,{{\rm{l}}}{{\rm{o}}}{{\rm{b}}}{{\rm{e}}}}-{S}_{0,{{\rm{l}}}{{\rm{o}}}{{\rm{b}}}{{\rm{e}}}})/{S}_{1,{{\rm{l}}}{{\rm{o}}}{{\rm{b}}}{{\rm{e}}}}$$

where *S*_0, lobe_ is the mean signal intensity in a lobe before contrast injection and *S*_1, lobe_ is the signal intensity at all post-injection image acquisition points [18]. The maximum slope of the wash-in period of the time-intensity curve of each liver lobe was used as a measure for the perfusion rate [19]. The relative perfusion rate was compared with the relative number of microspheres delivered to each liver lobe.

#### Qualitative analysis: fictive dose maps

The Ho-sensitive T2*-weighted scans facilitated MRI-voxel-based dosimetry using a fictional specific activity of 12 MBq/mg. The scans were processed in Q-suite (version 2.2, Quirem Medical B.V.) to calculate dose maps for visual assessment of homogeneity.

#### Quantitative analysis: homogeneity indices

The dose maps were then loaded into custom Matlab acquisition code to calculate the homogeneity index (HI)

$${{\rm{H}}}{{\rm{I}}}=({D}_{2{{\rm{ \% }}}}-{D}_{98{{\rm{ \% }}}})/{D}_{{{\rm{m}}}{{\rm{e}}}{{\rm{a}}}{{\rm{n}}}}$$

where *D*_2%_ is the near maximum, *D*_98%_ the near minimum, and *D*_mean_ the mean dose [20]. A lower HI represents an increased dose homogeneity.

#### Quantitative analysis: dose distribution per liver lobe

After generating dose maps for the whole-liver, dose maps for each individual liver lobe (left lateral lobe, left medial lobe, right medial lobe, right lateral lobe and caudate lobe, later referred to as lobes 1 to 5, respectively [21]) were obtained as well to gain deeper insights into spatial variations in dose distribution patterns.

#### Ho microsphere distribution following high dosages

To address the secondary aim of our study, to assess microsphere distribution patterns following the administration of large numbers of microspheres to a porcine liver, four additional fractions of 1,000 mg ^165^Ho microspheres were administered to six of the nine included livers.

#### Micro-computed tomography (micro-CT) analysis

Tissue samples (20 × 20 × 20 mm) were obtained from each liver lobe with a disposable scalpel (Swann-Morton, Owlerton Green). The samples were fixed in phosphate-buffered 4% formalin (Merck KGaA) for seven days. Three-dimensional high-resolution images were acquired using a micro-CT system (IRIS PET/CT, Inviscan SAS) with a spatial resolution of 60 µm. Other imaging parameters included: tube voltage = 80 kVp; exposure = 0.56 mAs; slice thickness = 0.029 mm. The data were reconstructed with a voxel size of 30 µm. A segmentation threshold of 350 HU was applied.

### Statistical analysis

Continuous variables were summarised as mean ± standard deviation for normally distributed data and median with interquartile range (IQR) for skewed data. To determine the correlation between perfusion rate and microsphere distribution, the Pearson’s correlation coefficient (*r*) was used, with 0 ≤ *r* < 0.1 none, 0.1 ≤ *r* < 0.3 poor, 0.3 ≤ *r* < 0.6 fair, 0.6 ≤ *r* < 0.8 moderate, 0.8 ≤ *r* < 1 very strong, *r* = 1 perfect correlation [22]. The data were processed using SPSS statistical software package version 29 (SPSS Inc.).

## Results

### Liver procurement and investigated blood flow rates

The median liver weight before perfusion was 2,637 g (IQR 2,496–2,671 g). Median weight gain after perfusion was +15.59% (IQR 9.54–16.73%). To achieve HA flow rates of 0.02, 0.15 and 0.22 mL/min/g liver tissue, corresponding mean HA pump settings of 51 ± 1.15, 440 ± 85.44, and 583 ± 15.28 mL/min were required. Two livers were cannulated directly via the HA, and all other livers via an aortic cannula.

### Image analysis

#### Perfusion rates

**Fig. 2:**
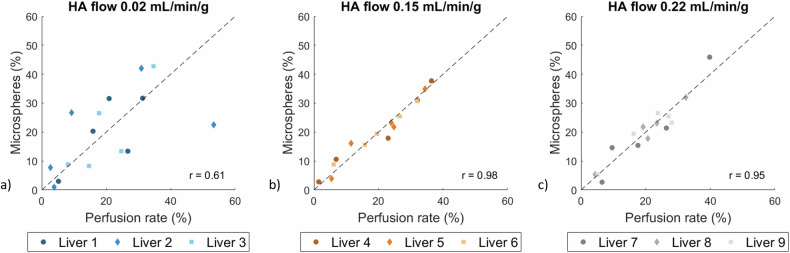
Perfusion rate and number of microspheres per liver lobe for *ex vivo* porcine livers under various HA blood flow rates of (**a**) 0.02, (**b**) 0.15, and (**c**) 0.22 mL/min/g liver tissue. The dashed line represents a perfect correlation. HA, Hepatic artery; *r*, Pearson’s correlation coefficient

The dynamic contrast-enhanced MRI scans revealed small regions of reduced arterial perfusion for livers from all HA blood flow rate categories, typically affecting one or two liver lobes. Nonetheless, a moderate correlation between perfusion rate and the number of microspheres per liver lobe was observed at low HA flow rates (*r* = 0.611, *p* = 0.015), increasing to a very strong correlation at higher HA flow rates (*r* = 0.977, *p* < 0.001 and *r* = 0.951, *p* < 0.001 for 0.15 and 0.22 mL/min/g, respectively) (see Fig. 2).

#### Qualitative analysis: fictive dose maps

**Fig. 3:**
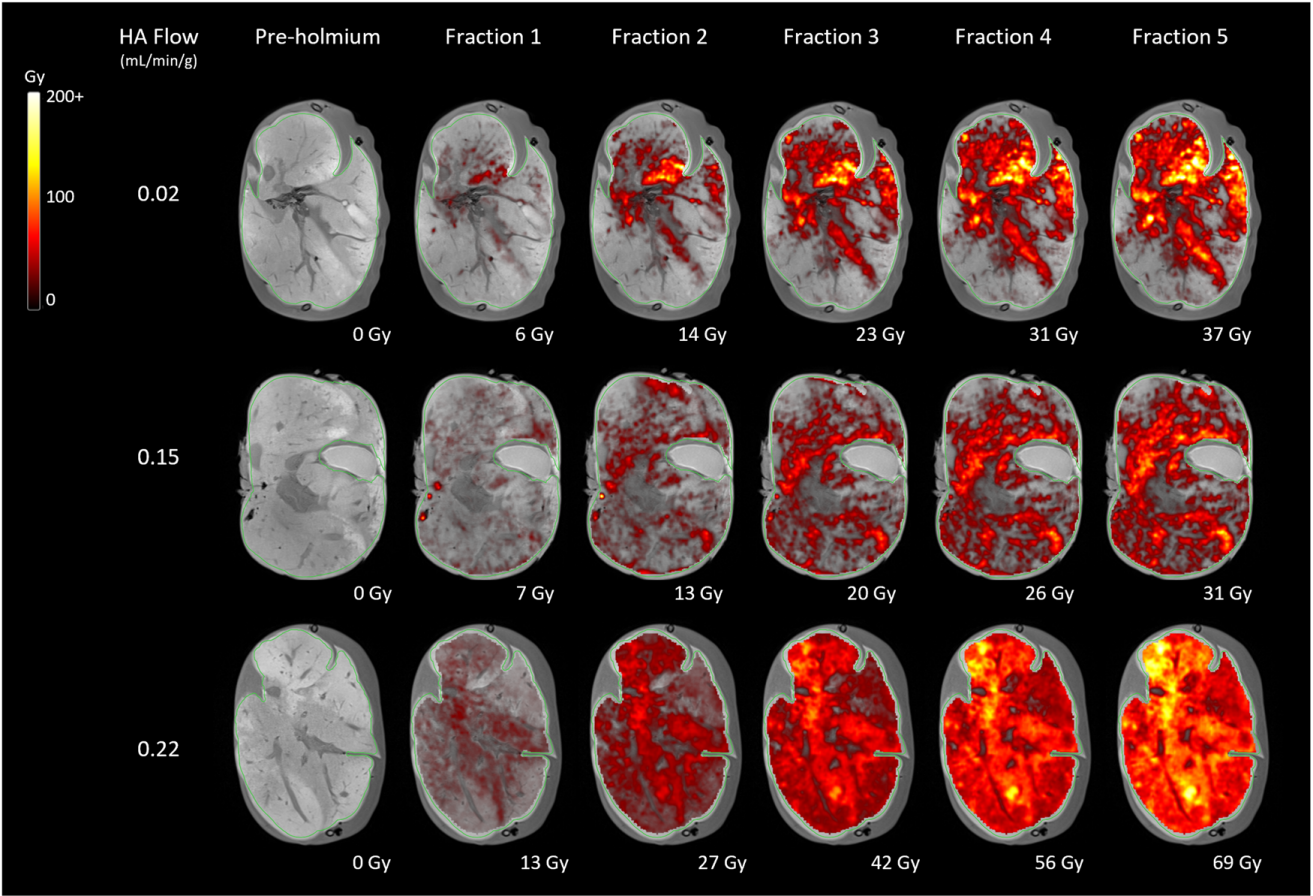
MRI-based dose maps of three *ex vivo* porcine livers after administering multiple fractions of microspheres under various HA blood flow rates. All dose maps were scaled from 0 to 200 Gy. Gy, Gray; HA, Hepatic artery; MRI, Magnetic resonance imaging

**Fig. 4:**
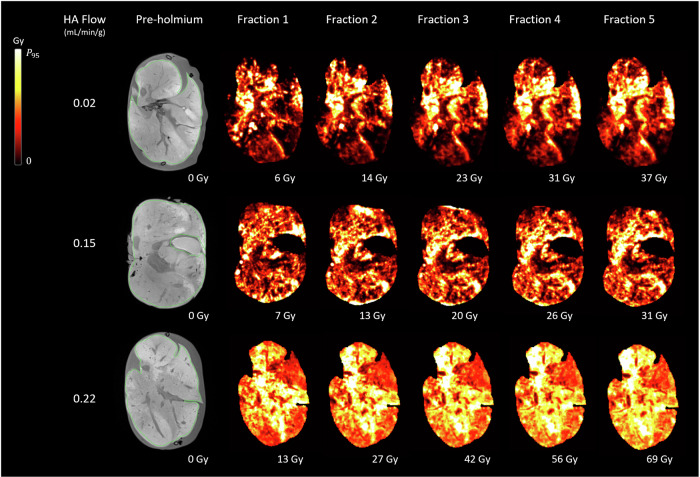
MRI-based dose maps of three *ex vivo* porcine livers after administering multiple fractions of microspheres under various HA blood flow rates. All dose maps were scaled to a maximum corresponding to the 95th percentile value. Gy, Gray; HA, Hepatic artery; MRI, Magnetic resonance imaging

Representative examples of MRI-based dose maps of the porcine livers perfused at various HA flow rates can be found in Fig. 3 (scaled to a maximum value of 200 Gy) and Fig. 4 (scaled to a maximum corresponding to the 95th percentile value). The MRI-based dose maps of all nine porcine livers are available in Supplemental #3. Both figures show a more clustered distribution pattern at low HA flow rates, resulting in greater heterogeneity compared to higher HA flow rates. Figure 4 shows that microsphere clusters from fraction 1 expanded with subsequent fractions, suggesting that administering 20% of the dose predicts the distribution of subsequent fractions.

#### Quantitative analysis: homogeneity indices

**Fig. 5:**
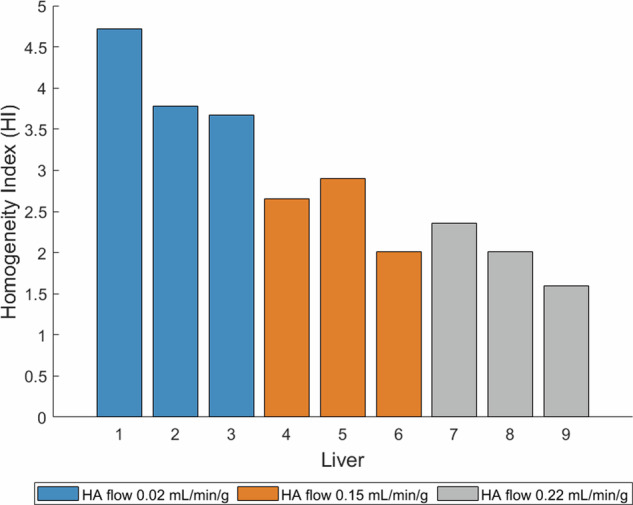
Homogeneity index of the MRI-based dose maps after administering five fractions of 250 mg microspheres under various HA blood flow rates. HA, Hepatic artery; MRI, Magnetic resonance imaging

The HIs (Fig. 5) for the three investigated flows correspond to the visually observed dose distribution patterns; the HI of the dose map was high at low HA flow rates, indicating a more heterogeneous dose distribution pattern, whereas the HI of the dose maps was low at high HA flow rates, indicating a more homogeneous dose distribution pattern. At an intermediate HA flow rate, the dose maps displayed both homogeneous and heterogeneous distribution patterns upon visual observation.

#### Quantitative analysis: dose distribution per liver lobe

**Fig. 6:**
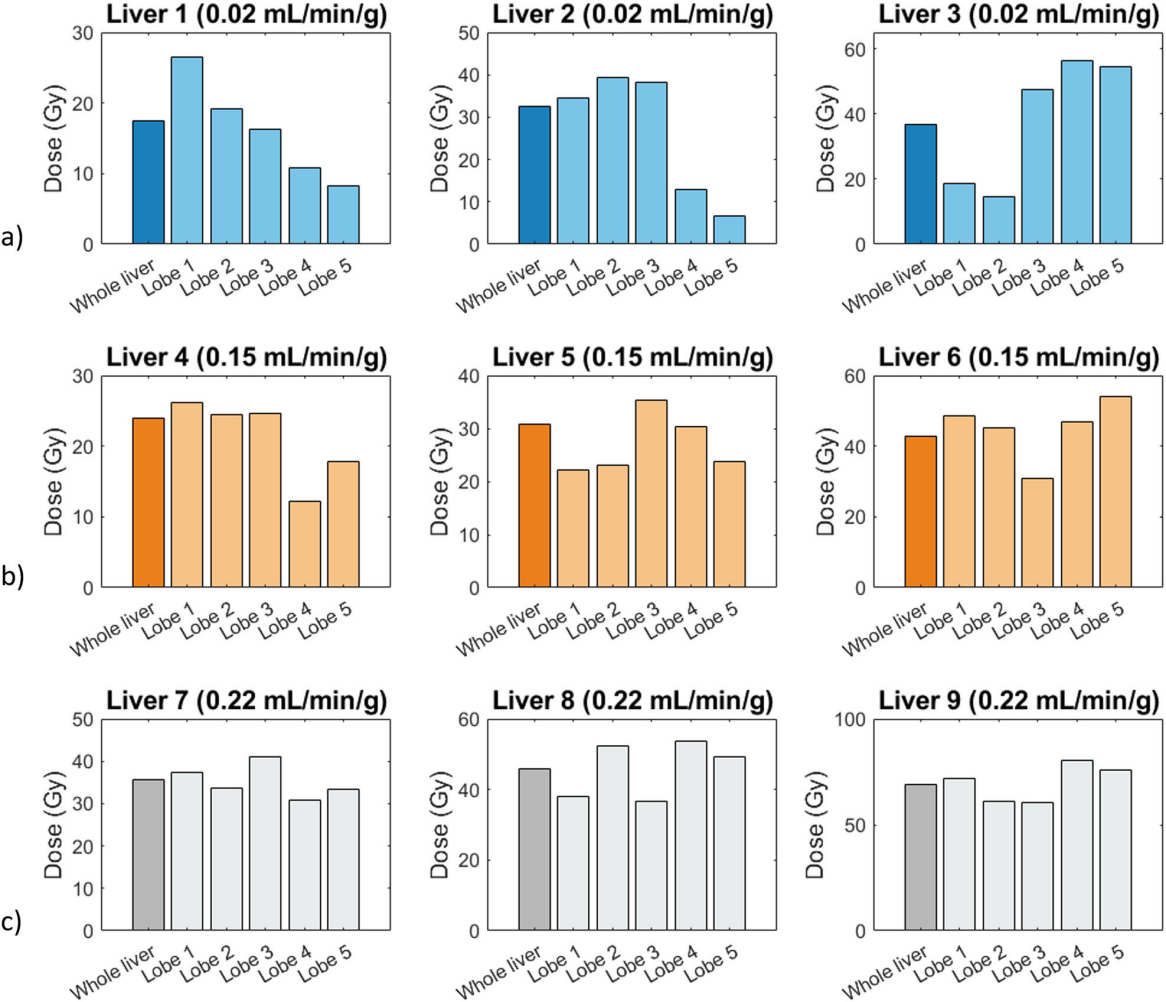
Dose per liver lobe in Gy after administering five fractions of 250 mg microspheres under various HA blood flow rates of (**a**) 0.02, (**b**) 0.15, and (**c**) 0.22 mL/min/g liver tissue. Lobe 1: left lateral lobe; lobe 2: left medial lobe; lobe 3: right medial lobe; lobe 4: right lateral lobe; lobe 5: caudate lobe; note that the y-axis differs per graph. Gy, Gray; HA, Hepatic artery

Dose maps for each liver lobe can be found in Fig. 6. Particularly under low and medium flow conditions, certain liver lobes received lower doses than others. This variation influenced the HI, as lower dose delivery inherently resulted in more heterogeneous distributions.

#### Ho microsphere distribution following high dosages

**Fig. 7:**
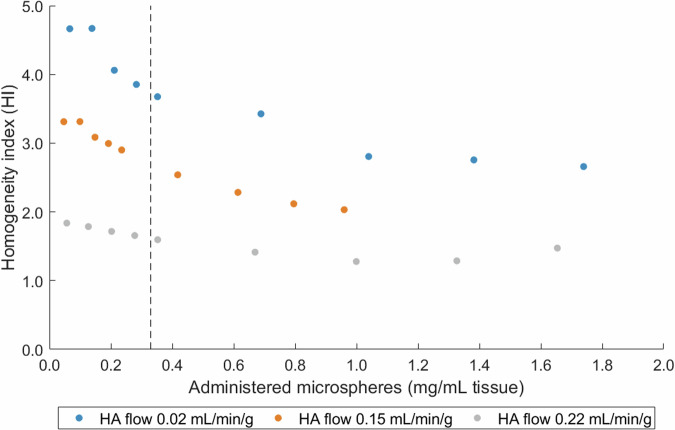
Scatterplot of the homogeneity index against the number of administered microspheres (in mg per mL liver tissue) under various HA blood flow rates, for three livers to which four extra fractions of 1,000 mg microspheres were administered. A lower HI indicates a more homogeneous distribution. The dashed line represents the commonly used clinical dose limit of 60 Gy for whole-liver absorbed dose [25, 44]. HA, hepatic artery; HI, Homogeneity index

The administration of four additional fractions of 1,000 mg microspheres resulted in a mean total mass of 4,498 ± 341 mg microspheres (1.45 ± 0.11 mg microspheres per mL of tissue) delivered to the porcine livers. Residual microspheres remaining in the V-vial were accounted for in this calculation. Overall distribution patterns did not alter (Supplemental #4, Fig. S2). The microsphere concentration continued to increase in a relatively linear manner with each successive fraction (Supplemental #4, Fig. S3). Arterial pressure also increased with each injected fraction, with the most pronounced increase at the high HA flow rate (Supplemental #4, Fig. S4). The HI decreased as the concentration of administered microspheres increased (Fig. 7). The plateau observed after administering more than 1.00 mg of microspheres per mL of tissue suggests that homogeneity does not continue to increase indefinitely.

#### Micro-CT analysis

**Fig. 8:**
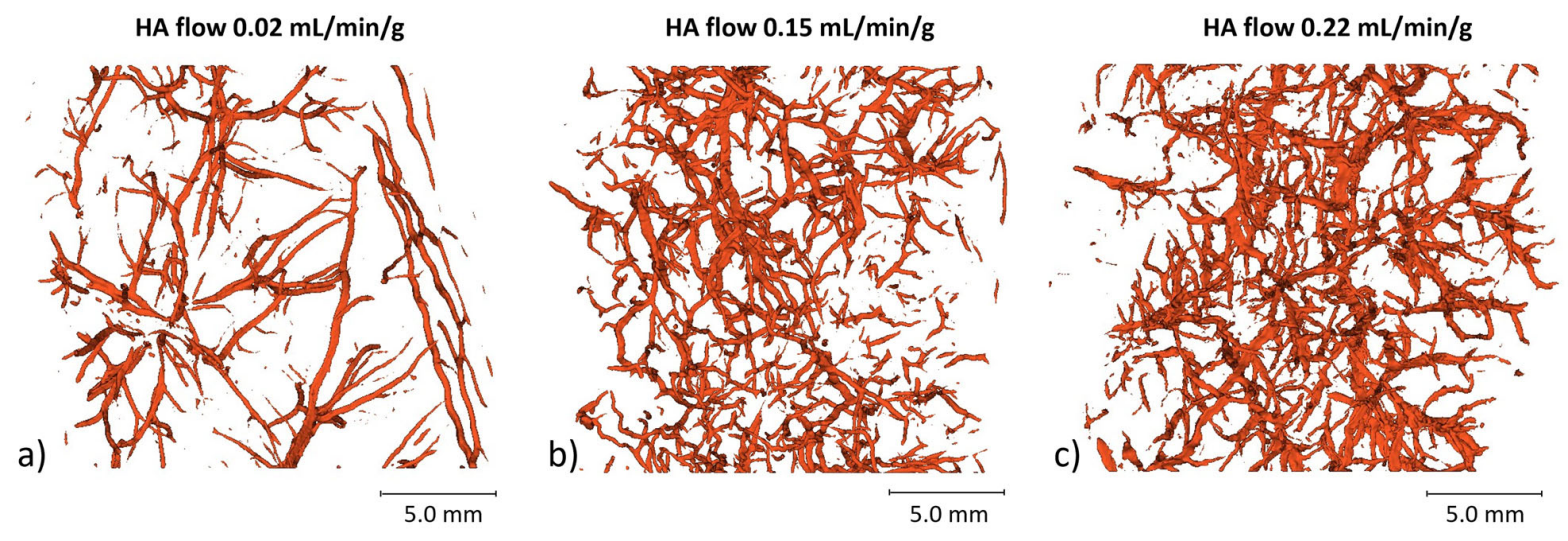
Three-dimensional micro-computed tomography reconstructions of ^165^Ho microspheres lodging in the HA vasculature for tissue samples perfused at a HA blood flow rate of (**a**) 0.02 mL/min/g liver tissue, (**b**) 0.15 mL/min/g liver tissue, and (**c**) 0.22 mL/min/g liver tissue. The respective mass of microspheres administered to these livers was 1.74, 1.75, and 1.65 mg microspheres per mL of liver tissue. HA, hepatic artery

Figure 8 presents representative micro-CT reconstructions of tissue samples from livers perfused at various HA flow rates. The reconstructions reveal that at high HA flow rate, a larger number of smaller vessels are filled with microspheres. In contrast, at low HA flow rate, microsphere distribution is predominantly limited to larger vessels, consistent with observations from the macroscopic MRI data.

## Discussion

This explorative study suggests that a higher HA flow rate leads to more homogeneous microsphere distributions compared to a lower HA flow rate, where more heterogeneous microsphere distribution patterns were observed.

The HA flow rate is highly variable in diseased livers (220–813 mL/min [8, 9]). Moreover, flow to normal liver tissue is considerably variable between segments and also within one segment [23]. According to the results from this study, this might result in different microsphere distribution patterns. A possible explanation lies in the degree of mixing between microspheres and blood under various HA blood flow rates. The perfusion rate *versus* number of microspheres per liver lobe (Fig. 2) suggests that higher HA flow rate improves mixing, as indicated by the strong correlation between perfusion rate and number of microspheres. In contrast, a lower HA flow rate reduces mixing efficiency, causing microspheres to deviate from the contrast agent flow. Dynamic contrast-enhanced MRI has shown effectiveness in indicating tissue perfusion [19, 24], supporting the assumption that contrast agent distribution can predict blood flow. Although factors such as injection rate and contrast bolus dispersion influence the absolute quantification of blood flow, their effect on relative blood flow, as used in this study, is assumed to be minimal.

The injection rate was maintained far above the investigated blood flow rates, taking vessel diameters into account. Microsphere injection velocity was 188 cm/s through a catheter with a 0.6 mm inner diameter, while blood flow velocities were 4 and 50 cm/s for low and high HA flow rate, respectively, assuming a HA diameter of 5 mm. Consequently, the injection-to-blood flow velocity ratio was highest under low HA flow rate conditions. Previous studies demonstrated that higher microsphere injection rates enhance the mixing of microspheres with blood [6, 7]. Aramburu et al [6] suggested that a higher ratio between microsphere injection rate and blood flow rate resulted in more microspheres crossing blood flow streamlines, though our study suggests that this effect may be mitigated if the blood flow rate is too low.

The administration of large numbers of microspheres (mean total mass of 4,498 ± 341 mg), did not significantly alter distribution patterns. This was contrary to the expectation that an increased particle load would cause stasis and backflow [10]. Microsphere uptake remained relatively linear (Supplemental #4, Fig. S3). This raises the question of whether clinically observed backflow is primarily caused by a high microsphere load. A more plausible explanation for stasis and backflow could be vasospasms. This issue might be better addressed by administering a vasodilator rather than reducing the number of microspheres while increasing their specific activity. However, the number of microspheres administered may still have been below the maximum threshold. In the clinic, a whole-liver absorbed dose limit of 60 Gy is maintained. When scaled to the median porcine liver weight, this corresponds to an administration of 0.33 mg microspheres per mL of tissue, which was greatly exceeded in this study (1.45 mg/mL). However, higher doses can be delivered to a single lobe or segment, as long as the average whole-liver absorbed dose does not exceed 60 Gy [25]. During superselective TARE targeting one lobe (about 1/5 of the liver), a dose of 300 Gy could be administered, corresponding to a microsphere concentration of 1.64 mg/mL. This value is slightly higher than the median dose administered in this study. Future experiments could explore the effects of stasis and backflow by overfilling a single lobe or segment.

The micro-CT reconstructions showed that higher HA flow rates resulted in a greater number of smaller vessels being filled, whereas lower HA flow rates primarily filled larger vessels. This aligned with the macroscopic MRI observations, as the preferential filling of larger vessels for low HA flow rates resulted in a more clustered distribution pattern. Under these conditions, microspheres may settle more proximally in the vasculature, potentially obstructing the pathway to smaller vessels, which is undesirable for achieving a homogeneous distribution within the tumour.

In current clinical practice, the HA blood flow rate is unknown before a TARE procedure. However, it is a variable that can be adjusted, for example, by administering angiotensin II. Since tumour cells lack adrenergic innervation [26], the infusion of angiotensin II constricts normal arterial vessels while leaving tumour vessels largely unaffected. This results in reduced blood flow to healthy liver tissue, while maintaining or even increasing flow to the tumour vasculature [27]. The increased flow towards tumour tissue may promote more homogeneous distribution patterns within the tumour, while the reduced flow to healthy liver tissue may lead to decreased and more heterogeneous distributions in non-tumourous areas. Hemingway et al [28] showed for small metastases (2 cm) increased perfusion up to ten times its baseline value within 130–240 s after the start of angiotensin II injection. However, for larger tumours, the effect was minimal. In addition, angiotensin II interacts with other physiological systems, complicating its isolated use [29].

Another way to influence the HA blood flow rate is by using an anti-reflux or balloon catheter, which can decrease blood pressure in the downstream compartments [30, 31]. This approach can approximate the low HA flow rate condition tested in this study (0.02 mL/min/g liver tissue). A reduction in total blood flow rate is again favourable for healthy liver tissue by promoting a more heterogeneous distribution, while it has proven to increase the number of microspheres towards targeted areas as described by Rose et al [31].

During superselective TARE, the catheter is advanced deeper into the liver vasculature, resulting in microsphere release under lower HA flow rate conditions compared to lobar or whole-liver TARE. Our findings indicate that low HA flow rates promote a more heterogeneous microsphere distribution. Awareness of this phenomenon is important, and further research could investigate the differences in microsphere distribution patterns between superselective and whole-liver TARE at specific HA flow rates.

During this study microsphere injection rate was controlled using syringe pumps. In clinical practice, microspheres are manually administered, making it challenging to maintain a consistent injection rate, while previous studies have shown that injection rate influences microsphere distribution [7, 32]. Additionally, the catheter was positioned at least two centimetres before the bifurcation into the left and right HA, minimising the influence of the bifurcation on the distribution [33]. However, the radial catheter position could not be controlled, despite its known influence on microsphere distribution [34].

Furthermore, utilising slaughterhouse porcine livers offers benefits, such as reduced regulatory requirements, operational costs and ethical burden compared to performing live animal surgery. Porcine livers were preferred over livers from other species due to their similarities to human livers in terms of vascularity [21]. However, each porcine liver exhibits distinct anatomical characteristics, including variation between HA diameters, resulting in distinct physiological behaviours and making direct comparisons within the three HA flow rate groups challenging.

Several limitations must be acknowledged. First, the small sample size. Only three livers were included per flow rate. Second, only the effect of HA flow rate on healthy liver parenchyma was studied. Literature suggests that microspheres distribute heterogeneously in both healthy and tumourous liver tissue [35, 36]. However, unlike normal liver tissue, tumourous tissue has an unstructured architecture and altered blood flow patterns due to neovascularisation [37], which may influence microsphere deposition. Therefore, future studies would benefit from investigating the effects of HA flow rate on microsphere distribution in tumour-bearing livers.

Another limitation is that only Ho-loaded microspheres (30 µm, 1.4 g/mL) were studied. An *in vitro* comparison between resin and glass microspheres demonstrated that resin microspheres (29 µm, 1.6 g/mL), which are quite similar in size and density to Ho microspheres, exhibited a significantly greater penetration depth than glass microspheres (25 µm, 2.5 g/mL) in a planar tumour model [38]. Investigating the impact of microsphere properties such as size, density, and composition in this *ex vivo* setup would be a valuable direction for future research.

Izamis et al [11] reported technical challenges in *ex vivo* perfusion of slaughterhouse porcine livers, such as achieving a flush time within 30 min of warm ischaemia and managing PV air emboli. Similar issues were encountered in our study. Immediate cannulation of the PV reduced air emboli. However, air emboli within the PV, and to a lesser extent within the HA, remained visible on MRI. As these emboli appeared consistently in both pre-and post-Ho-sensitive T2*-weighted scans, they did not interfere with the image analysis. Similar to Izamis et al [11], our study required 17 livers before a reliable model was established and the experiments could be started. Our primary limitation was the blunt dissection from the aorta up to the point of division into five branches supplying each liver lobe, along with the ligation of all the branches. Significant leakage was frequently observed following cannulation and initiation of perfusion. Additionally, high pressures during perfusion complicated pump operation, mainly due to the extended tubing connecting the liver in the MRI room to the pump in the control room. Arens et al [39] reported that 1 metre ¼ inch tubing adds 11 mmHg resistance at a blood flow rate of 1 L/min.

The *ex vivo* environment in which this study was conducted may result in physiological responses that differ from those observed *in vivo*. *In vivo*, vascular reactivity could be expected during the administration of microspheres. Due to preferential HA blood flow towards tumour tissue, microsphere administration may saturate the tumour microvasculature, which could lead to flow redistributions and alterations in tumour-to-nontumour ratios during treatment. However, the EMERITUS-1 study by Roosen et al [40] found a linear correlation between absorbed tumour dose and injected activity in 78% of the cases, suggesting that most tumours were not saturated at the end of treatment. Similarly, our *ex vivo* study showed no saturation in healthy liver tissue, even after administering four additional fractions of 1,000 mg microspheres. Furthermore, the absence of organs with a clearance function could lead to acid-base imbalances and elevated glucose levels [41]. We deliberately chose not to compensate for these phenomena, which may have contributed to the gradual decline of the *ex vivo* livers over time. Our primary focus was to maintain patency of the liver vasculature and use the *ex vivo* livers solely as a vascular model. To assess the microsphere distribution, Ho-sensitive T2*-weighted scans acquired immediately before and after administering microsphere fractions were compared. The interval between these scans was only 5 min, during which deterioration of the liver was considered negligible.

Another potential limitation of the *ex vivo* porcine liver model is the presence of regions with reduced arterial perfusion, detectable on the dynamic contrast-enhanced MRIs as areas with delayed and less steep wash-in curves. Gravante et al [42] noted that reduced arterial perfusion commonly affects parts of the caudate, right lateral, and right part of the medial lobe, or a combination of these regions. However, no flat contrast curves—indicative of non-perfused areas—were observed in this study. Additionally, microspheres successfully lodged within the delayed perfusion regions. Alzaraa et al [43] found that such reduced perfusion regions, even after 4 h of perfusion, had no significant impact on flow rate, pressure and oxygen saturation. Therefore, the presence of reduced arterial perfusion areas may have a minimal impact on the findings of this specific study.

While this study provides a first step in investigating the influence of HA flow rate on microsphere distribution, further research is needed to translate these findings to clinical practice. This could involve *in vivo* studies, correlating pre-TARE HA flow rate measurements with microsphere distributions and consequent treatment outcomes, or *in silico* studies, simulating different HA flow rates and their impact on patient-specific microsphere distributions.

To conclude, this study showed that a higher HA flow rate results in a more homogeneous microsphere distribution in *ex vivo* perfused healthy porcine livers. This is likely attributable to improved mixing between microspheres and blood under high HA flow rate conditions. However, low HA flow rate conditions seem to reduce the mixing effect. Moreover, no stasis or backflow was observed after the administration of large numbers of microspheres. Considering the high variation in HA flow rates between patients and the potential to adjust flow rate in clinical practice, for example, by using angiotensin II and different catheter types, investigating the impact of HA blood flow rate on tumourous tissue could be valuable to understand the influence of this patient-specific parameter on TARE.

## Supplementary information

**Additional file 1: Supplementary Table S1**. Flow rates and pressure settings for the hepatic artery and portal vein for *ex vivo* porcine liver studies. **Supplementary Fig. S1.** MRI-based dose maps of three *ex vivo* porcine livers after administering multiple fractions of microspheres under various HA blood flow rates. **Supplementary Fig. S2.** MRI-based dose maps of three *ex vivo* porcine livers after administering multiple fractions of microspheres under various HA blood flow rates. **Supplementary Fig. S3.** Scatterplot of the mean dose in the liver (in Gray) against the number of administered microspheres (in mg per mL of liver tissue) under various HA blood flow rates. **Supplementary Fig. S4**. Development of the arterial pressure after administering fractions of microspheres to the *ex vivo* porcine livers under various HA blood flow rates.

## Data Availability

The datasets used and/or analysed during the current study are available from the corresponding author on reasonable request.

## Abbreviations

*D*_2%_: Near maximum dose
*D*_98%_: Near minimum dose
*D*_mean_: Mean dose
HA: Hepatic artery
HI: Homogeneity index
IQR: Interquartile range
Micro-CT: Micro-computed tomography
MRI: Magnetic resonance imaging
PV: Portal vein
*S*_0, lobe_: Mean signal intensity in a lobe before contrast injection
*S*_1, lobe_: Signal intensity at all post-injection image acquisition points
TARE: Transarterial radioembolisation
