# Supplementary material for: The influence of hepatic arterial blood flow rate on holmium microsphere distribution: an MRI study in perfused porcine livers
Source: Eur Radiol Exp. 2025 Aug 6;9:69. doi: 10.1186/s41747-025-00609-7 (PMC12328857; doi:10.1186/s41747-025-00609-7)
Supplement: Supplementary file 1 — Additional file 1: Supplementary Table S1. Flow rates and pressure settings for the hepatic artery and portal vein for ex vivo porcine liver studies. Supplementary Fig. S1. MRI-based dose maps of three ex vivo porcine livers after administering multiple fractions of microspheres under various HA blood flow rates. Supplementary Fig. S2. MRI-based dose maps of three ex vivo porcine livers after administering multiple fractions of microspheres under various HA blood flow rates. Supplementary Fig. S3. Scatterplot of the mean dose in the liver (in Gray) against the number of administered microspheres (in mg per mL of liver tissue) under various HA blood flow rates. Supplementary Fig. S4. Development of the arterial pressure after administering fractions of microspheres to the ex vivo porcine livers under various HA blood flow rates. [file 41747_2025_609_MOESM1_ESM.pdf]

The influence of hepatic arterial blood flow rate on holmium microsphere distribution: an MRI study in perfused porcine livers

ELECTRONIC SUPPLEMENTARY MATERIAL

Supplemental #1 – Liver procurement protocol

The livers were procured from a local slaughterhouse. The process did not interfere with the standard abattoir protocol and was following the guidelines of the Dutch Food Safety Authority.

The complete organ package from the trachea to the rectum was requested and the liver was isolated from the surrounding organs, starting with the removal of the heart and lungs. The aorta and oesophagus were transected, and the oesophagus was secured with a ligature. [11] Subsequently, the portal venous was identified and cannulated with a 25-Fr cannula and the inferior vena cava was incised for outflow. Through the portal venous cannula, 1 L cold saline solution containing 5 mL 25,000 IE heparin was administered, followed by another 1.5 L cold saline solution. Ice was placed around the liver without direct contact. The interval between exsanguination and cold flush initiation was maintained under 30 min [11]. The liver was then isolated from the surrounding organs, starting with an incision between the pancreas and duodenum, extending towards the stomach until the oesophagus was reached. The spleen and kidneys were removed, and the abdominal aorta was identified. A large forceps was inserted in the aorta to secure the aorta and the hepatic artery. An incision from the other side of the pancreas along the bowels removed the remaining organs. The excised liver was placed in a transport bag and a portal venous flush of 0.5 L saline was initiated. The liver was packed in an insulated box with ice for transport to the laboratory.

Supplemental #2

Table S1. Flow rates and pressure settings for the hepatic artery and portal vein for *ex vivo* porcine liver studies.

First author [reference]	Hepatic arterial flow rate (mL/min/g)	Portal venous flow rate (mL/min/g)	Mean hepatic arterial pressure (mmHg)	Portal venous pressure (mmHg)	Mean liver weight (g)
Izamis [11]	0.1–0.35	0.77–1.35	60–90	9	-
Borie [45]	0.11–0.38 (25% of total hepatic flow)	0.34–1.15 (75% of total hepatic flow)	100–120	15–20	846
Adham [46]	0.25	0.73	81	13	-
Hickman [47]	0.09–0.14 (10–15% of total flow)	0.77–0.81 (85–90% of total flow)	-	-	-
Ikeda [48]	0.33	0.67	-	16	558
Mets [49]	0.14 (95/657)	0.65 (425/657)	<100	<18	657
Nagel [50]	0.18 (18/100)	0.42 (42/100)	80	8	1,277
Satoh [51]	0.25 (25% of total hepatic flow)	0.75 (75% of total hepatic flow)	80–120	7–15	-

Supplemental #3 – MRI-based dose maps

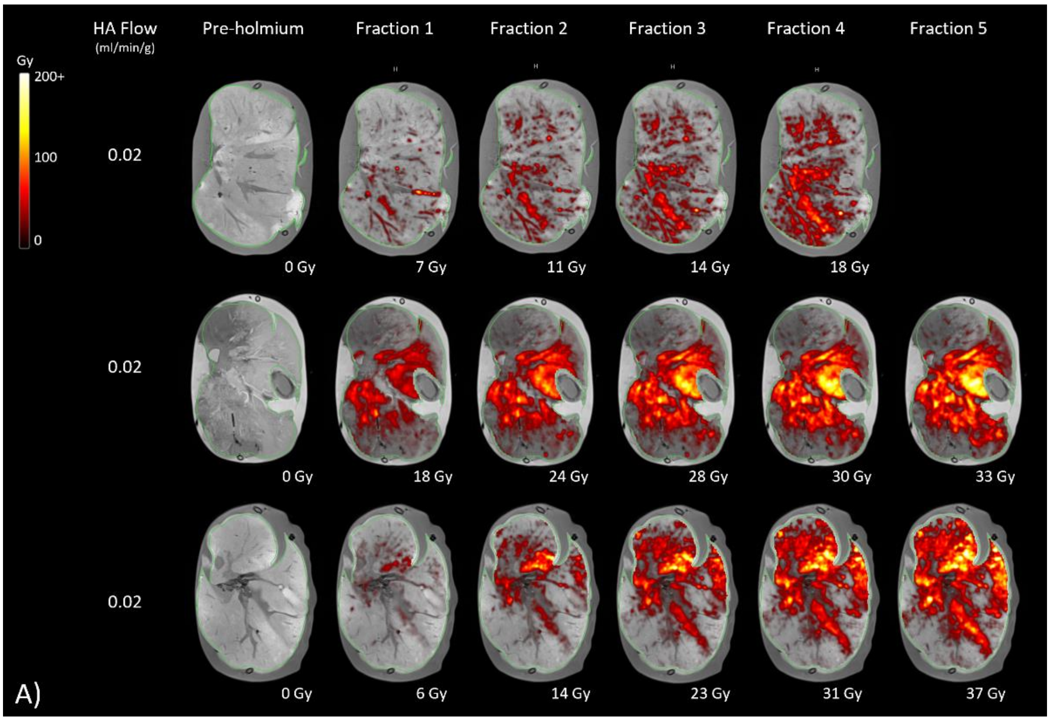

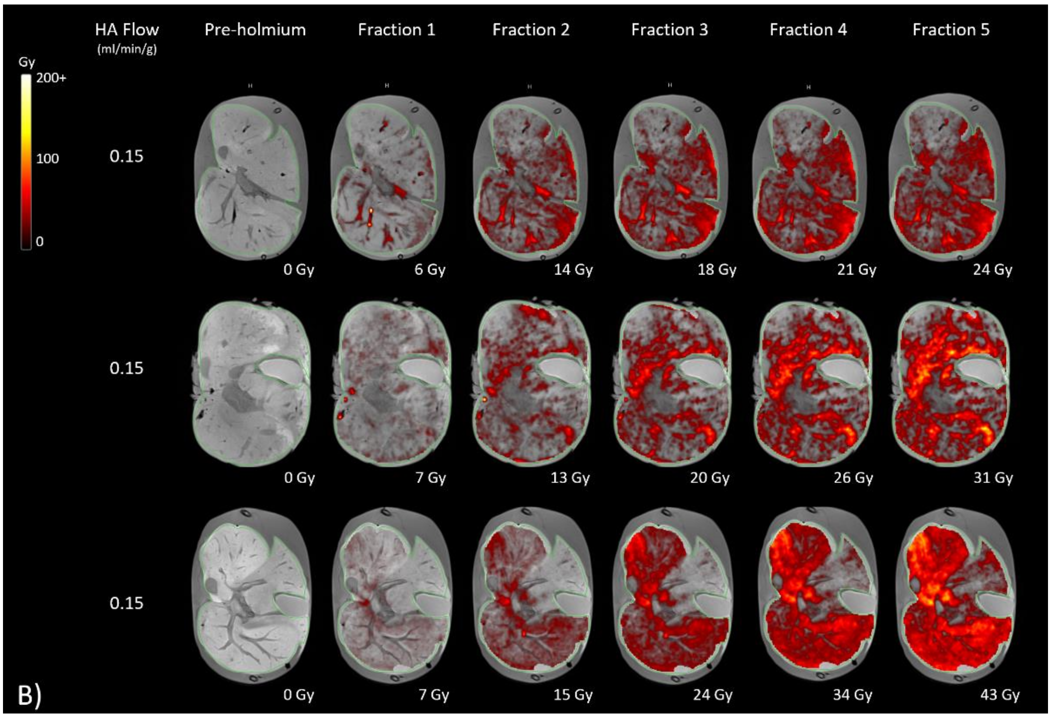

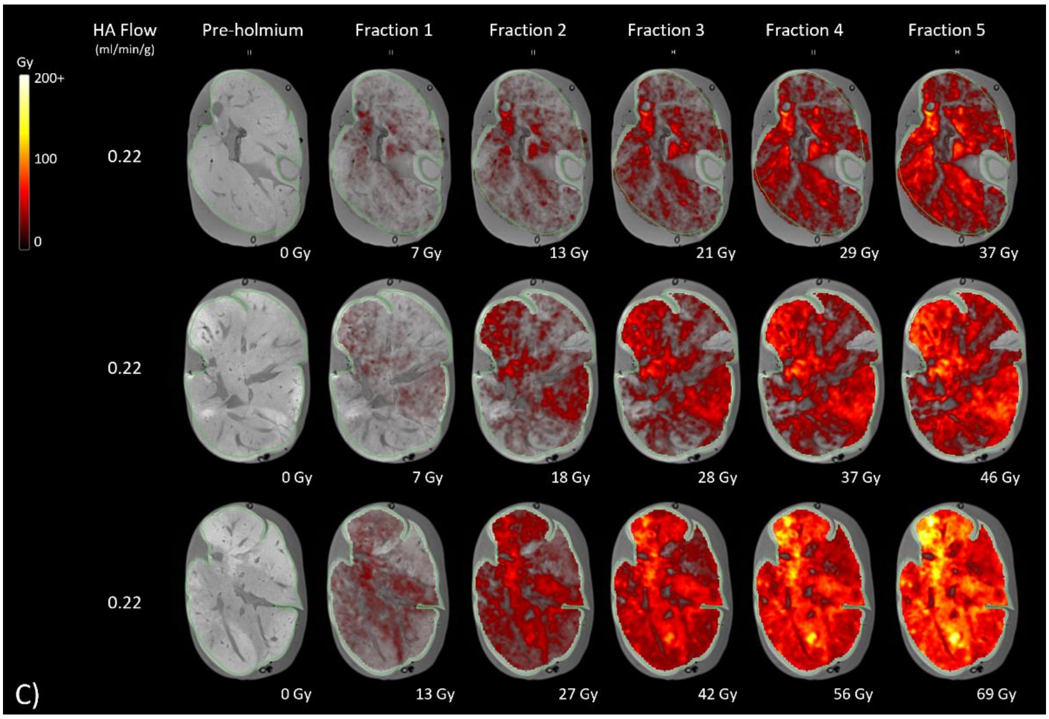

Fig. S1. MRI-based dose maps of three ex vivo porcine livers after administering multiple fractions of microspheres under various HA blood flow rates. (**a**) HA flow rate of 0.02 mL/min/g liver tissue. (**b**) HA flow rate of 0.15 mL/min/g liver tissue. (**c**) HA flow rate of 0.22 mL/min/g liver tissue. All dose maps were scaled from 0 to 200 Gy. *Gy* Gray, *HA* hepatic artery, *MRI* Magnetic resonance imaging.

Supplemental #4 – Administration of four extra fractions

Fig. S2 shows the resulting dose maps after administering four extra fractions of 1,000 mg ^{165}Ho microspheres after administration of the five regular fractions of 250 mg microspheres. In the scatterplot in Fig. S3, the mean dose after each fraction is plotted as a function of the number of microspheres that were administered and in Fig. S4 the development of the arterial pressure after administering several fractions of microspheres.

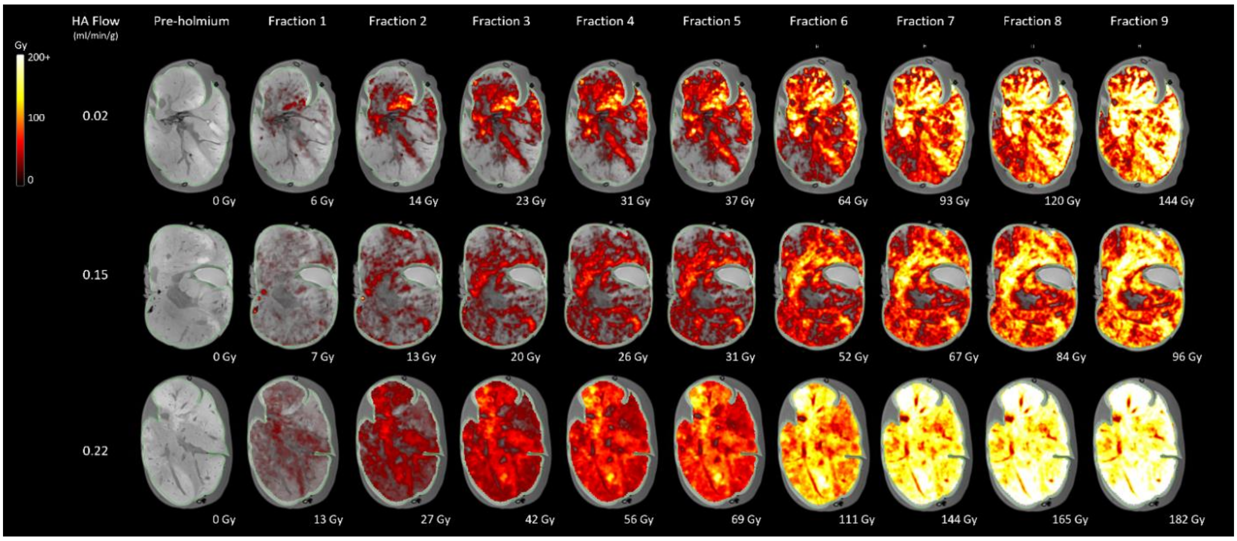

Fig. S2. MRI-based dose maps of three ex vivo porcine livers after administering multiple fractions of microspheres under various HA blood flow rates. First, five fractions of 250 mg microspheres were administered, followed by four fractions of 1,000 mg microspheres. All dose maps were scaled from 0 to 200 Gy. *Gy* Gray, *HA* hepatic artery, *MRI* Magnetic resonance imaging.

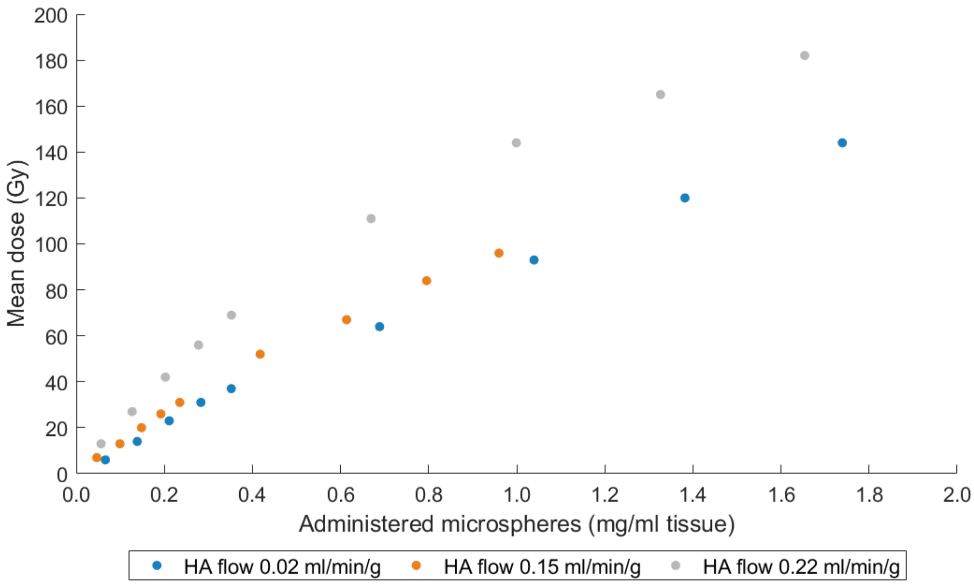

Fig. S3. Scatterplot of the mean dose in the liver (in Gray) against the number of administered microspheres (in mg per mL of liver tissue) under various HA blood flow rates. The microsphere concentration continued to increase relatively linearly with each successive fraction. Some flattening can be observed in the higher dose regions, potentially indicating that the maximum detectable MRI signal has been achieved. *Gy* Gray, *HA* Hepatic artery, *MRI* Magnetic resonance imaging.

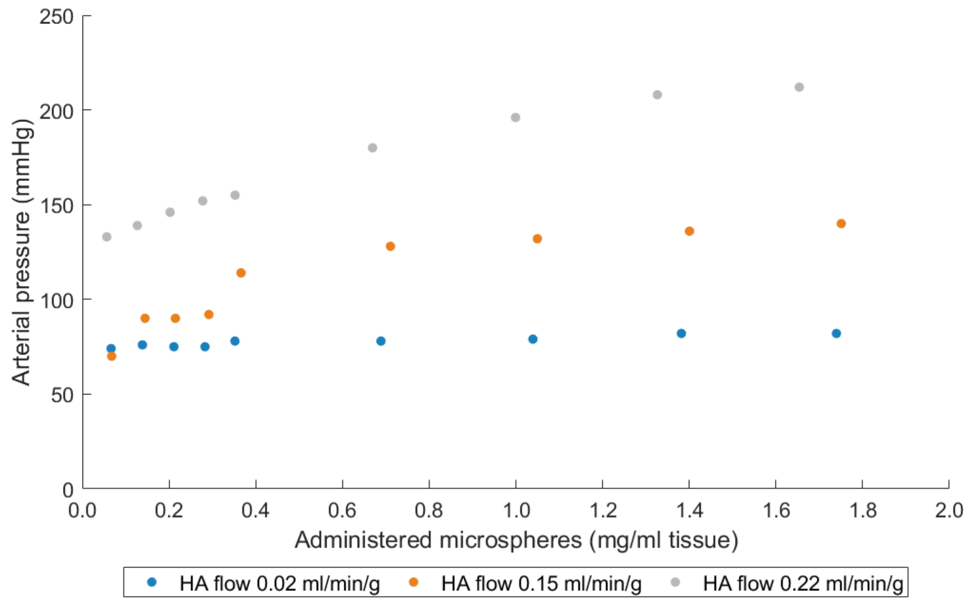

Fig. S4. Development of the arterial pressure after administering fractions of microspheres to the *ex vivo* porcine livers under various HA blood flow rates. During fraction 1–5, 250 mg microspheres per fraction was delivered, during fraction 6–9 1,000 mg. *HA* hepatic artery.

45 Borie DC, Eyraud D, Boleslawski E et al (2001) Functional metabolic characteristics of intact pig livers during prolonged extracorporeal perfusion: potential for a unique biological liver-assist device. Transplantation 72:393-405. Doi: https://doi.org/10.1097/00007890-200108150-00007

46 Adham M, Ducerf C, Roche EDL et al (1997) The isolated perfused porcine liver: assessment of viability during and after six hours of perfusion. Transplant International 10:299-311. Doi: https://doi.org/10.1111/j.1432-2277.1997.tb00708.x

47 Hickman R, Saunders SJ, Simson E, Terblanche J (2005) Perfusion of the isolated pig liver functional assessment under control normothermic conditions. British Journal of Surgery 58:33-38. Doi: https://doi.org/10.1002/bjs.1800580106

48 Ikeda T, Yanaga K, Lebeau G, Higashi H, Kakizoe S, Starzl TE (1990) Hemodynamic and biochemical changes during normothermic and hypothermic sanguinous perfusion of the porcine hepatic graft. Transplantation 50:564-567. Doi: https://doi.org/10.1097/00007890-199010000-00006

49 Mets B, Rose-Innes C, Lotz Z, Hickman R, Chalton D (1993) Comparison of in vivo and ex vivo porcine liver function using the same liver. J Hepatol 17:3-9. Doi: https://doi.org/10.1016/S0168-8278(05)80513-6

50 Nagel S, Hegemann O, Groneberg DA, Grosse-Siestrup C (2005) An improved model of isolated hemoperfused porcine livers using pneumatically driven pulsating blood pumps. Toxicologic Pathology 33:434-440. Doi: https://doi.org/10.1080/01926230590958164

51 Satoh S, Terajima H, Yagi T et al (1997) Humoral injury in porcine livers perfused with human whole blood. Transplantation 64:1117-1123. Doi: https://doi.org/10.1097/00007890-199710270-00006
